# Supplementary material for: Assaying Chlamydia pneumoniae Persistence in Monocyte-Derived Macrophages Identifies Dibenzocyclooctadiene Lignans as Phenotypic Switchers
Source: Molecules. 2020 Jan 11;25(2):294. doi: 10.3390/molecules25020294 (PMC7024427; doi:10.3390/molecules25020294)

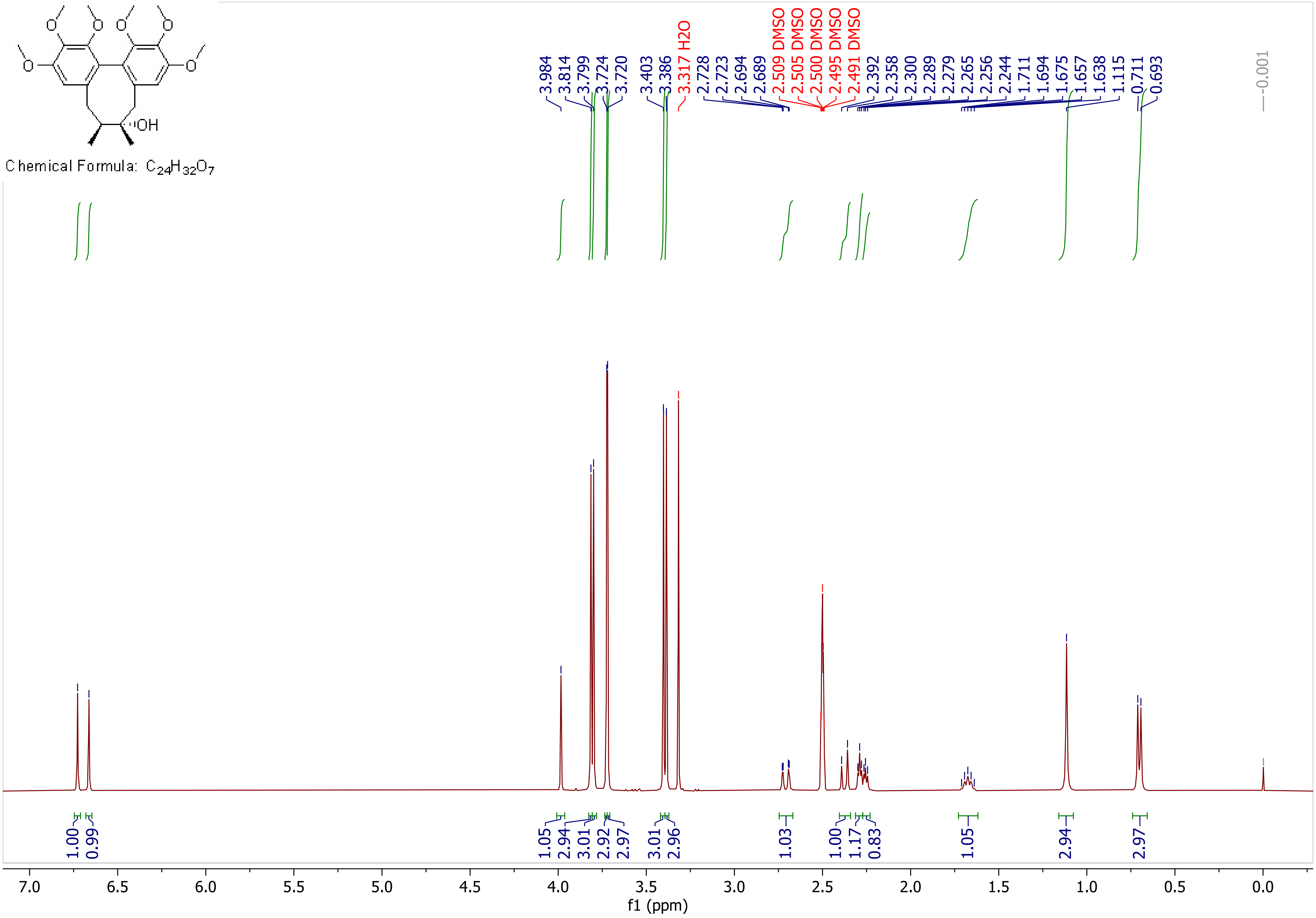

OH
O
Chemical Formula: C24H32O7
3.984
3.814
3.799
3.724
3.720
3.403
3.386
3.317 H2O
2.728
2.723
2.694
2.689
2.509 DMSO
2.505 DMSO
2.500 DMSO
2.495 DMSO
2.491 DMSO
2.392
2.358
2.300
2.289
2.279
2.265
2.256
2.244
1.711
1.694
1.675
1.657
1.638
1.115
0.711
0.693
-0.001
1.00
0.99
1.05
2.94
3.01
2.92
2.97
3.01
2.96
1.03
1.00
1.17
0.83
1.05
2.94
2.97
7.0
6.5
6.0
5.5
5.0
4.5
4.0
3.5
3.0
2.5
2.0
1.5
1.0
0.5
0.0
f1 (ppm)

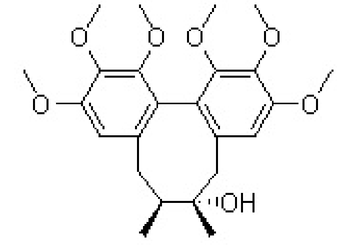

Chemical Formula: $C_{24}H_{32}O_7$

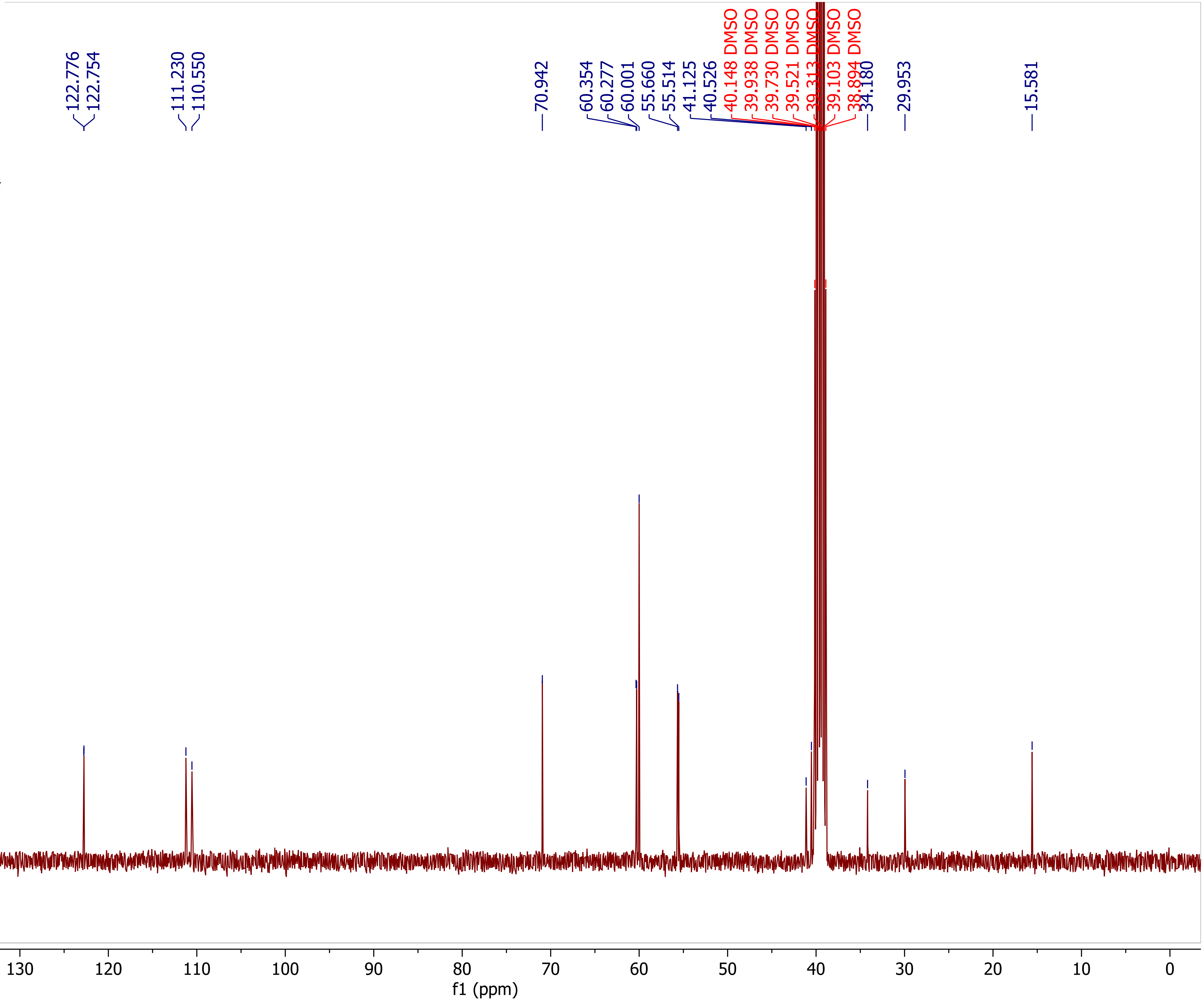

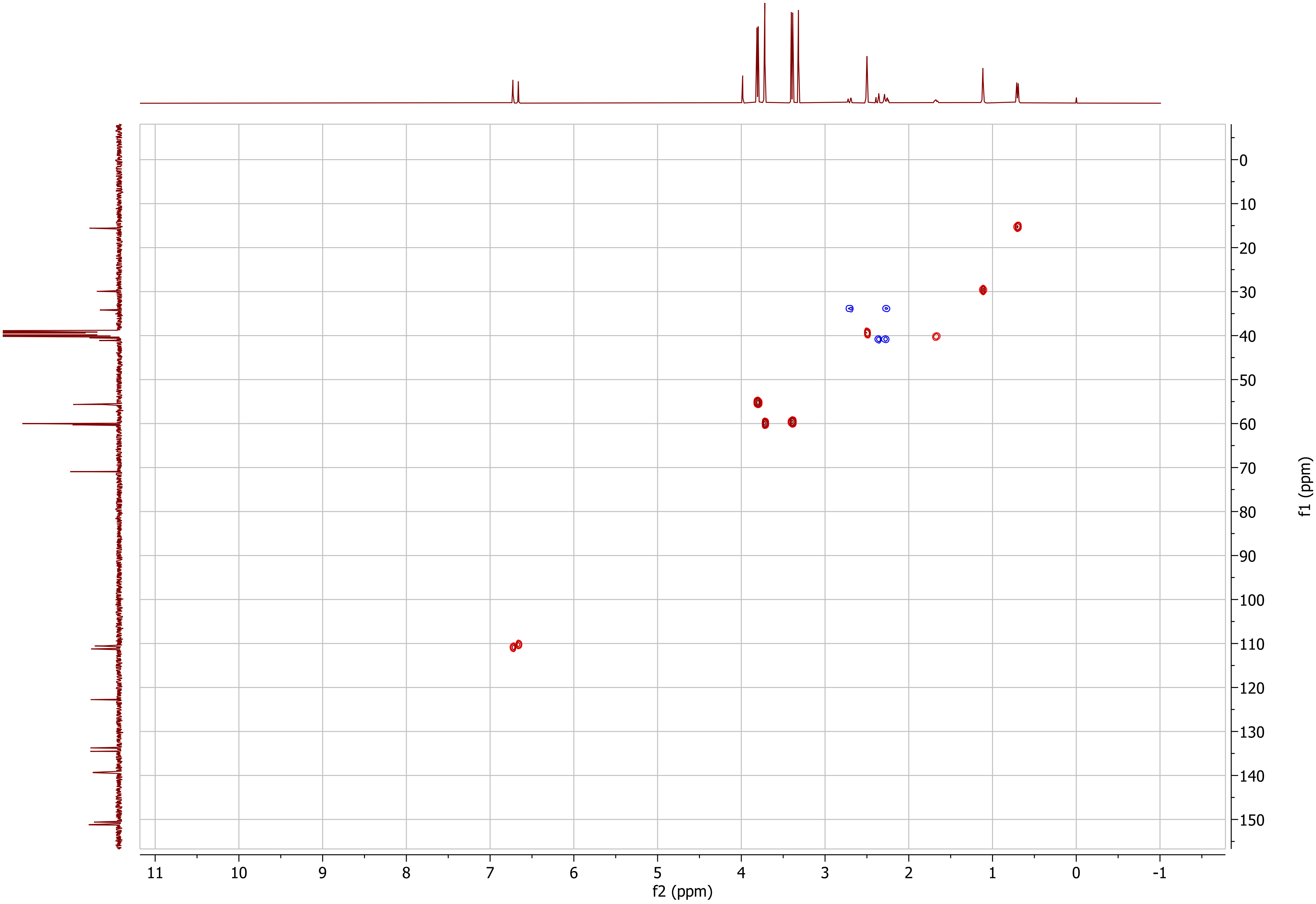

11
10
9
8
7
6
5
4
3
2
1
0
-1
f2 (ppm)
0
10
20
30
40
50
60
70
80
90
100
110
120
130
140
150
f1 (ppm)

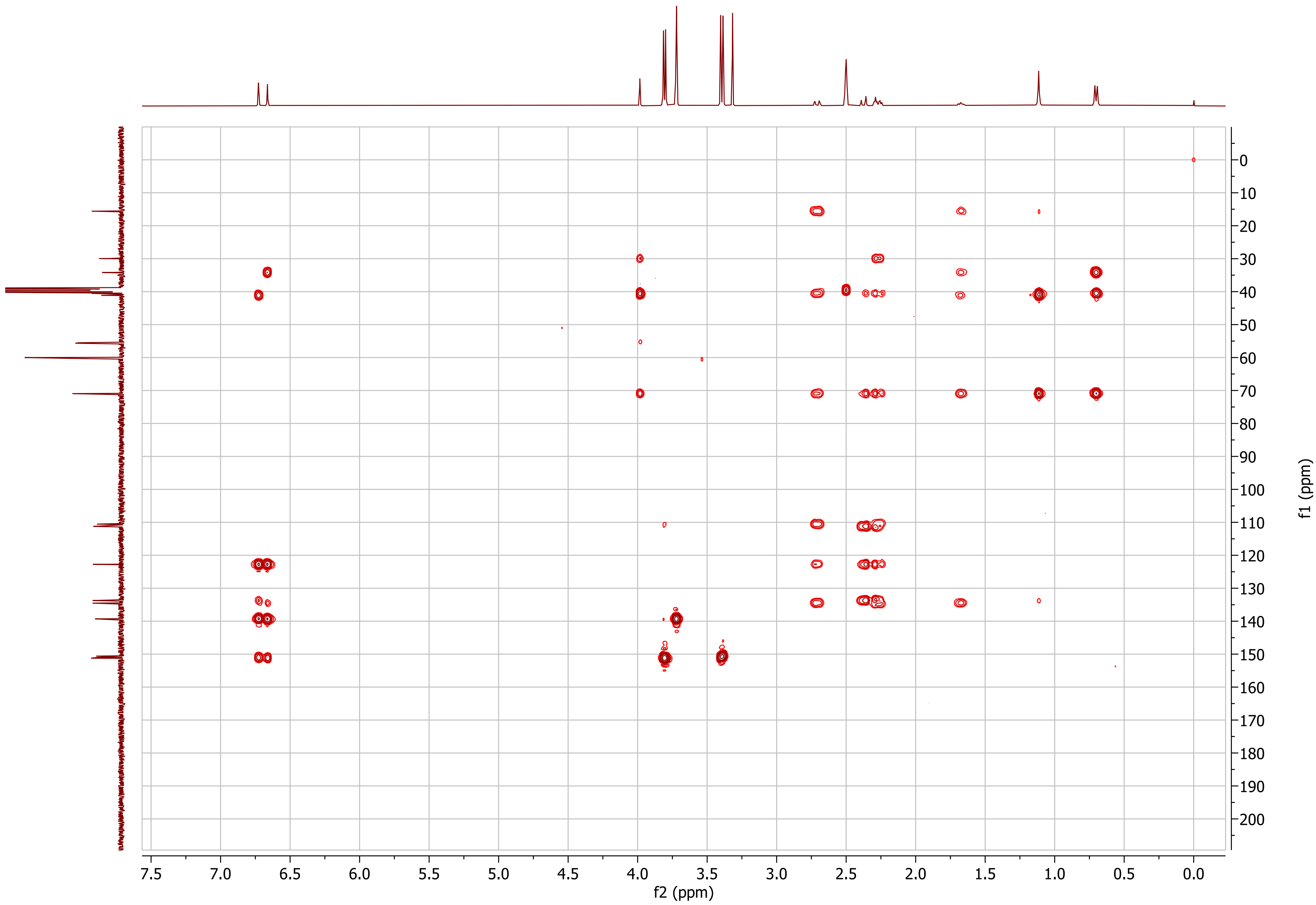

f2 (ppm)
f1 (ppm)
7.5
7.0
6.5
6.0
5.5
5.0
4.5
4.0
3.5
3.0
2.5
2.0
1.5
1.0
0.5
0.0
0
10
20
30
40
50
60
70
80
90
100
110
120
130
140
150
160
170
180
190
200

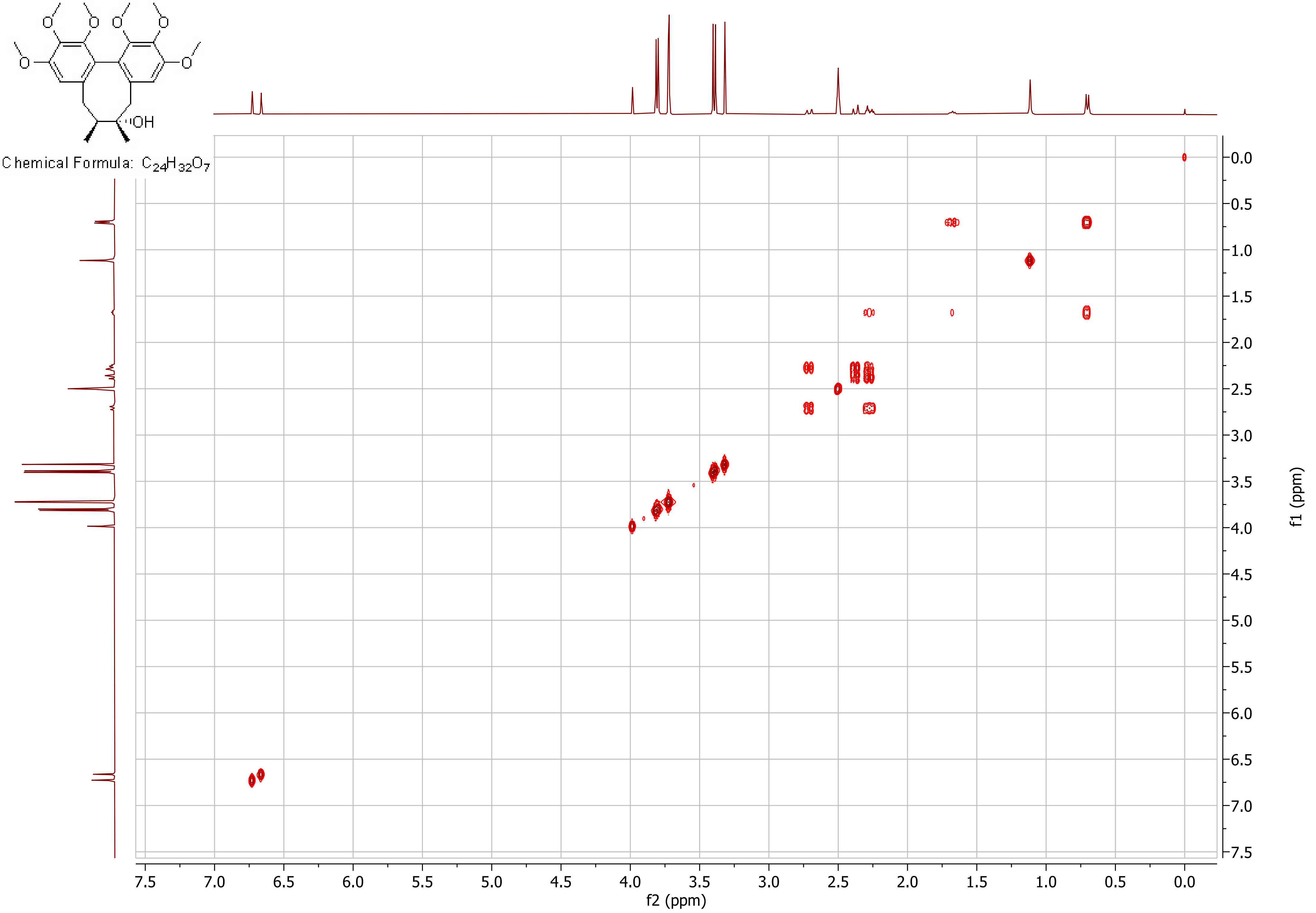
OH
Chemical Formula: $C_{24}H_{32}O_7$
f2 (ppm)
f1 (ppm)

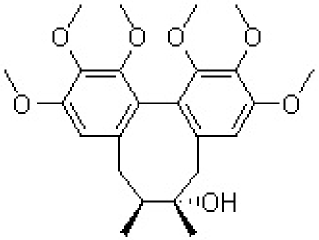

Chemical Formula: $C_{24}H_{32}O_7$

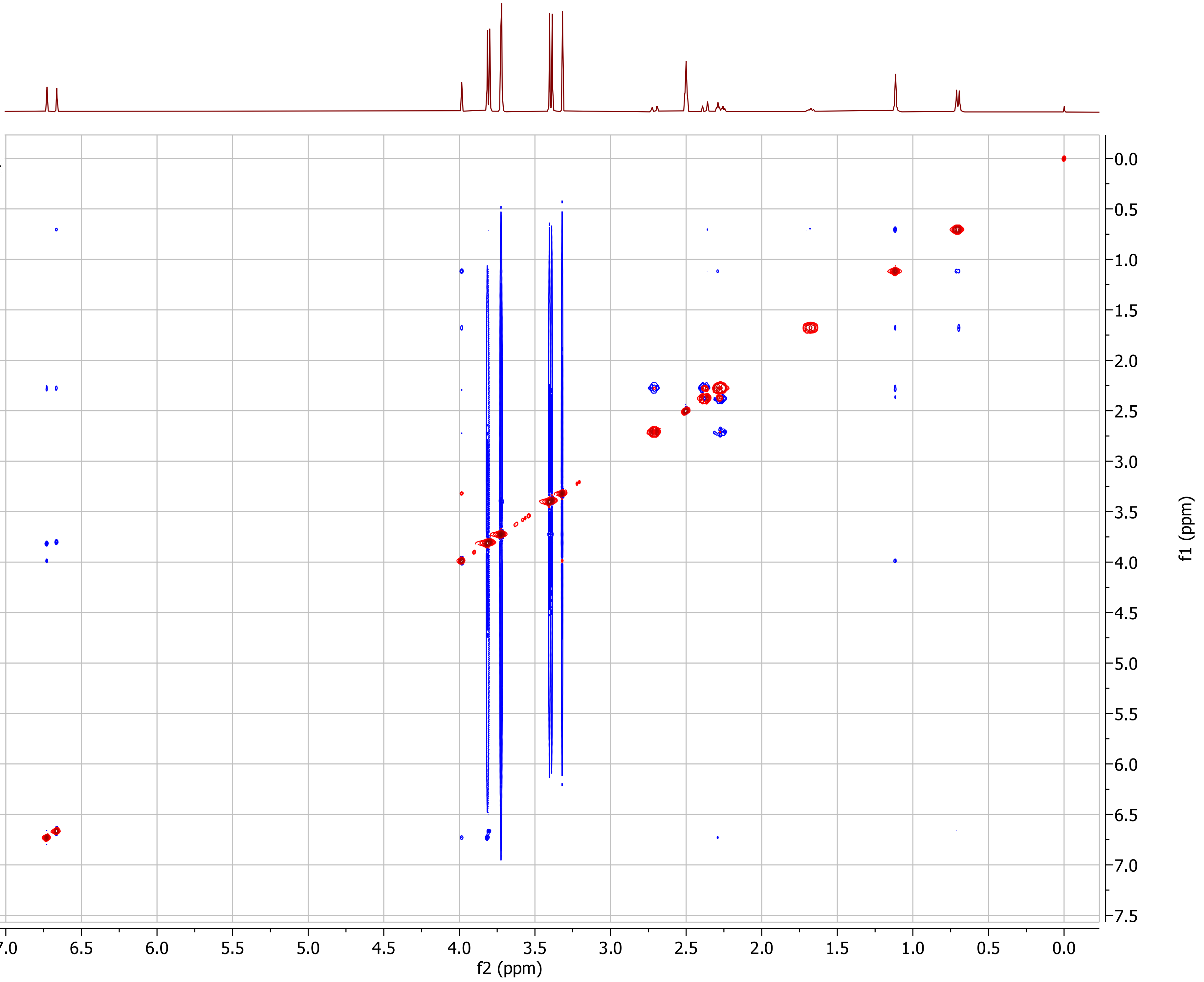

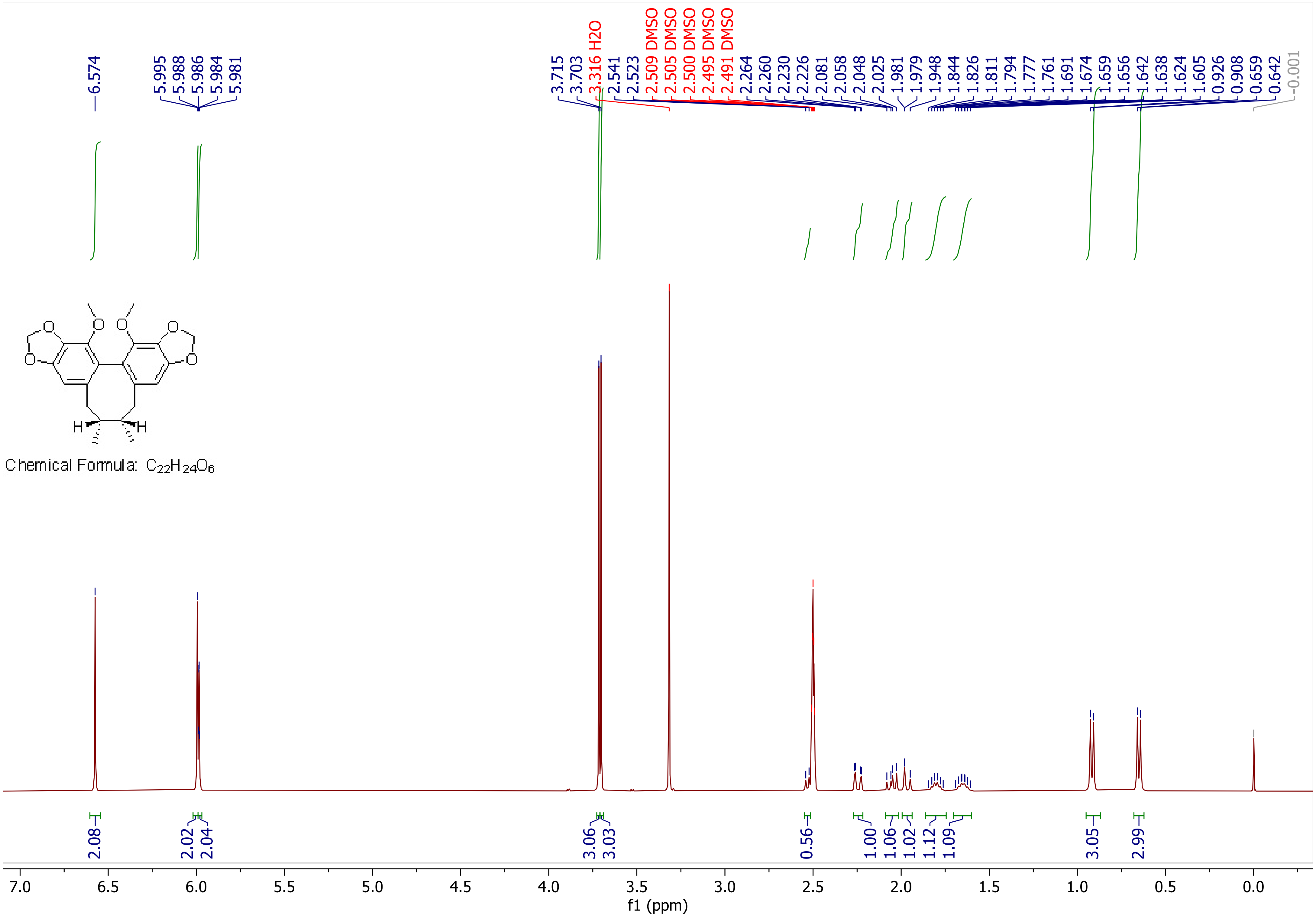
6.574
5.995
5.988
5.986
5.984
5.981
3.715
3.703
3.316 H2O
2.541
2.523
2.509 DMSO
2.505 DMSO
2.500 DMSO
2.495 DMSO
2.491 DMSO
2.264
2.260
2.230
2.226
2.081
2.058
2.048
2.025
1.981
1.979
1.948
1.844
1.826
1.811
1.794
1.777
1.761
1.691
1.674
1.659
1.656
1.642
1.638
1.624
1.605
0.926
0.908
0.659
0.642
-0.001
Chemical Formula: $C_{22}H_{24}O_6$
2.08
2.02
2.04
3.06
3.03
0.56
1.00
1.06
1.02
1.12
1.09
3.05
2.99
7.0
6.5
6.0
5.5
5.0
4.5
4.0
3.5
3.0
2.5
2.0
1.5
1.0
0.5
0.0
f1 (ppm)

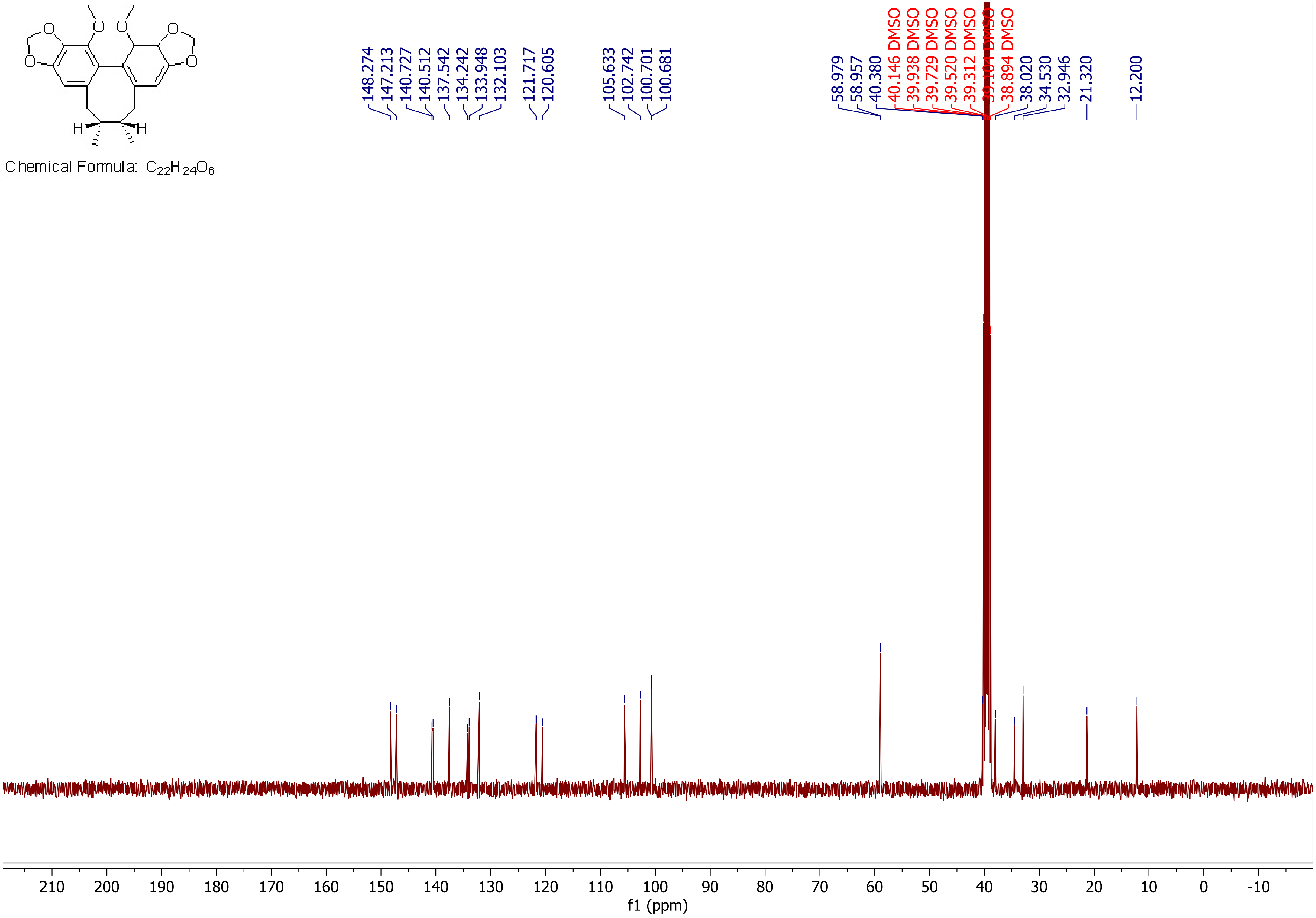

Chemical Formula: C22H24O6
148.274
147.213
140.727
140.512
137.542
134.242
133.948
132.103
121.717
120.605
105.633
102.742
100.701
100.681
58.979
58.957
40.380
40.146 DMSO
39.938 DMSO
39.729 DMSO
39.520 DMSO
39.312 DMSO
39.104 DMSO
38.894 DMSO
38.020
34.530
32.946
21.320
12.200
210 200 190 180 170 160 150 140 130 120 110 100 90 80 70 60 50 40 30 20 10 0 -10
f1 (ppm)

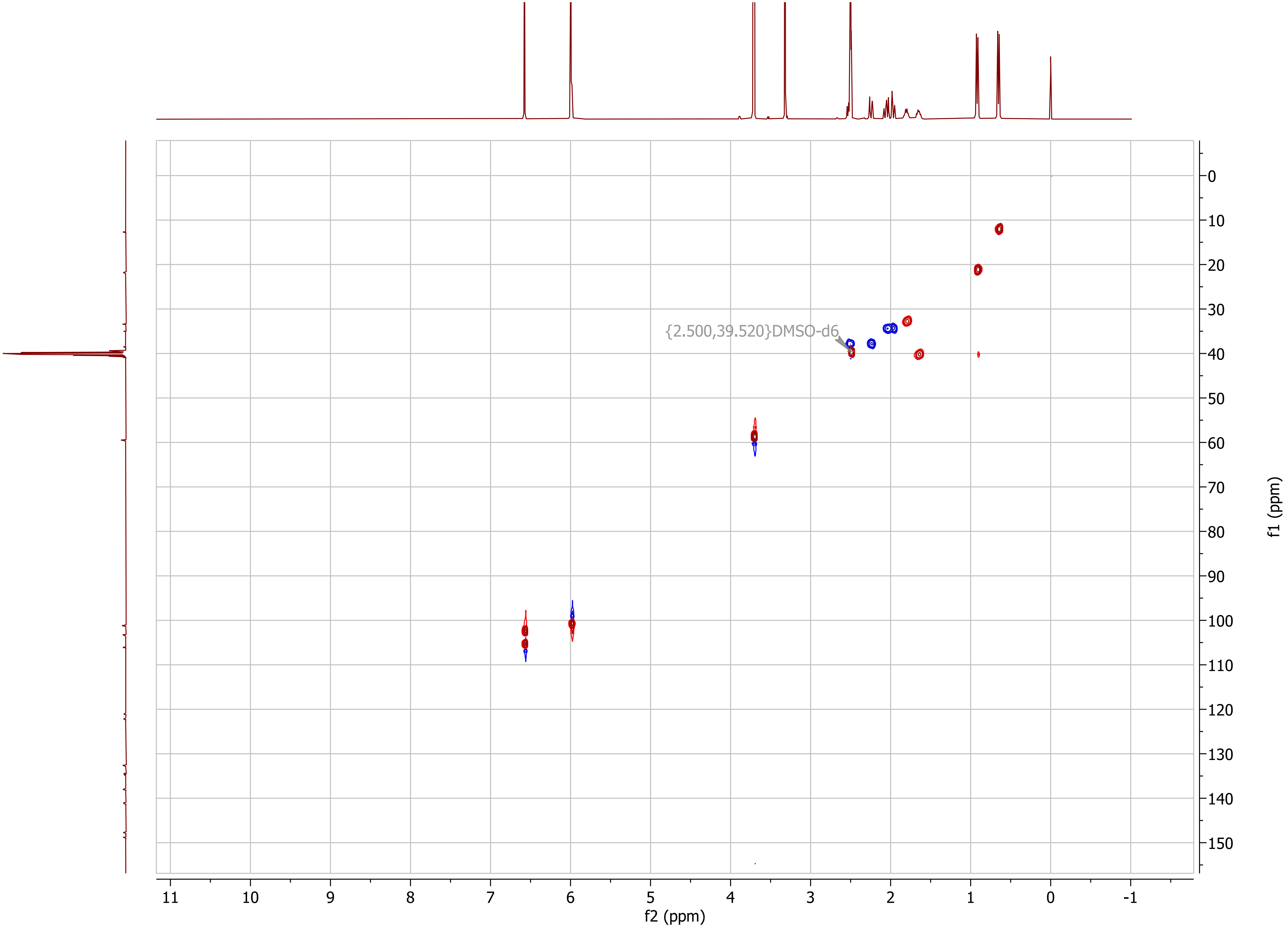

{2.500,39.520}DMSO-d6
f2 (ppm)
f1 (ppm)

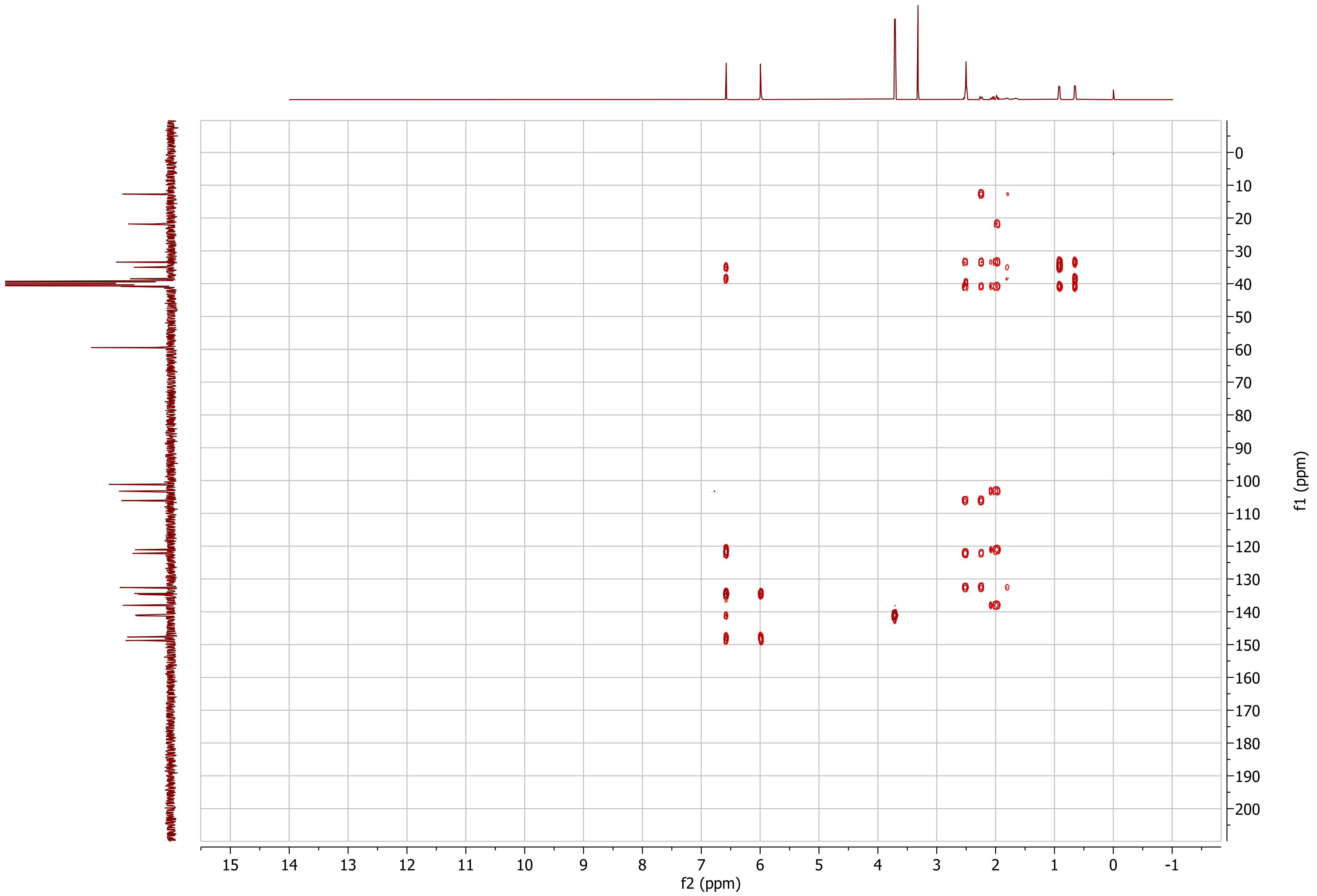

f2 (ppm)
f1 (ppm)

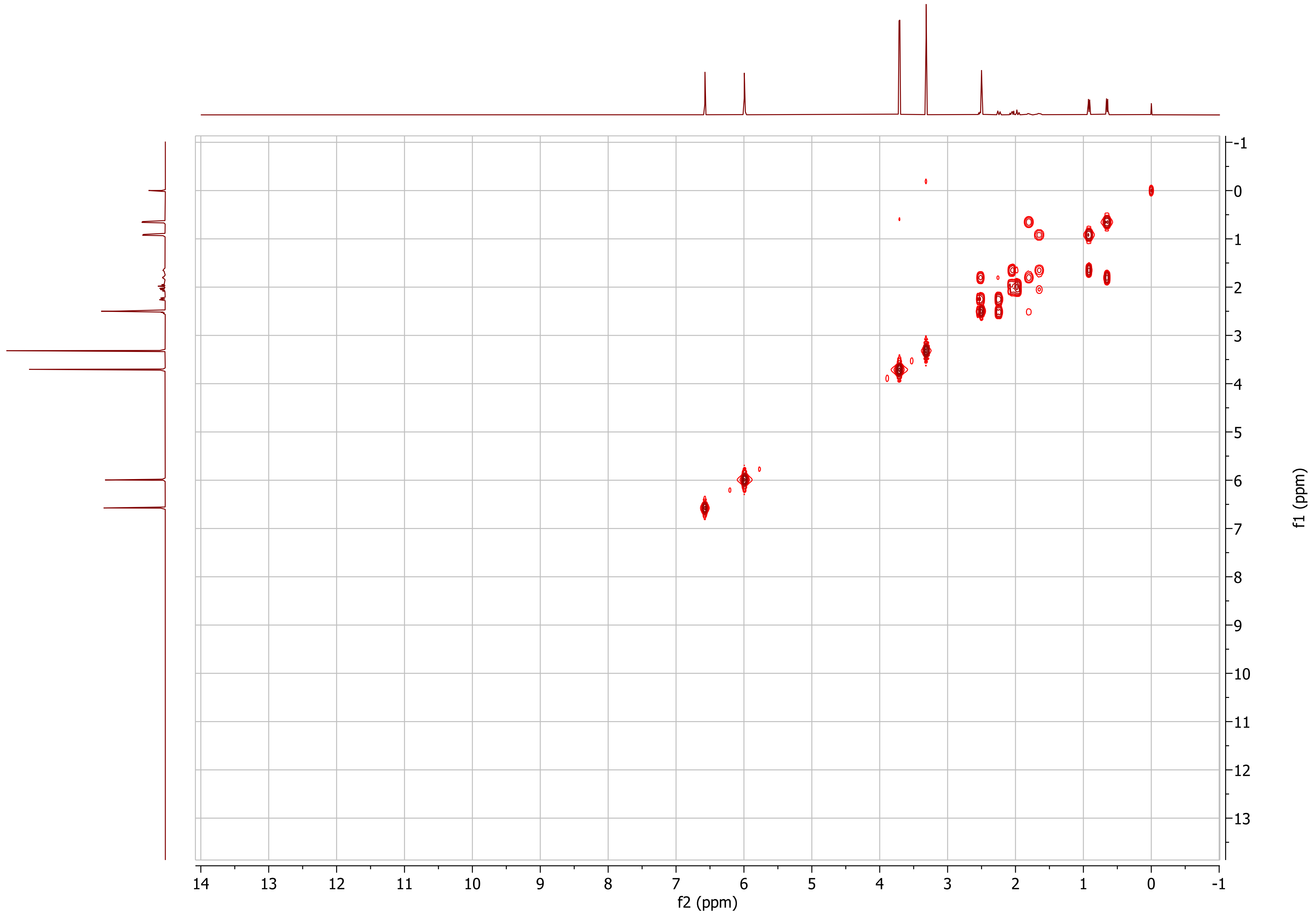
f2 (ppm)
f1 (ppm)

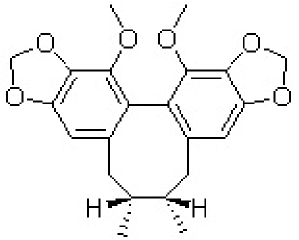

Chemical Formula: $C_{22}H_{24}O_6$

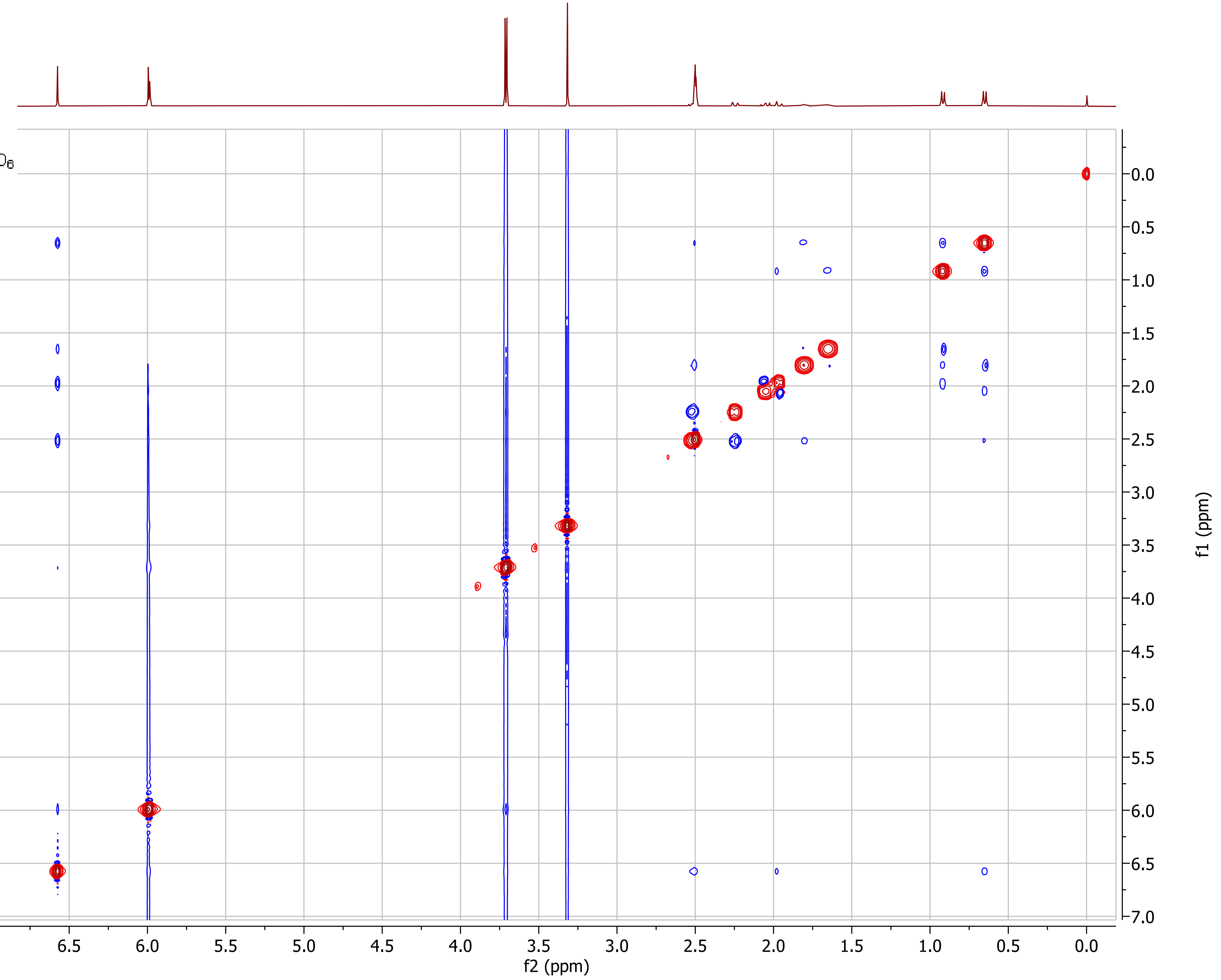

Supplement: Supplementary file 1 [file molecules-25-00294-s001.pdf]
